# Implications of mitochondrial membrane potential gradients on signaling and ATP production analyzed by correlative multi-parameter microscopy

**DOI:** 10.1038/s41598-024-65595-z

**Published:** 2024-06-26

**Authors:** Benjamin Gottschalk, Zhanat Koshenov, Roland Malli, Wolfgang F. Graier

**Affiliations:** 1 Molecular Biology and Biochemistry, Gottfried Schatz Research Center, Medical University of Graz, Neue Stiftingtalstraße 6/4, 8010 Graz, Austria; 2 BioTechMed, Graz, Austria

**Keywords:** Mitochondria, Mitochondrial membranes, Cristae junctions, Membrane potential gradient, Correlative microscopy, Biochemistry, Cell biology, Nanoscience and technology

## Abstract

The complex architecture and biochemistry of the inner mitochondrial membrane generate ultra-structures with different phospholipid and protein compositions, shapes, characteristics, and functions. The crista junction (CJ) serves as an important barrier separating the cristae (CM) and inner boundary membranes (IBM). Thereby CJ regulates the movement of ions and ensures distinct electrical potentials across the cristae (ΔΨ_C_) and inner boundary (ΔΨ_IBM_) membranes. We have developed a robust and flexible approach to visualize the CJ permeability with super-resolution microscopy as a readout of local mitochondrial membrane potential (ΔΨ_mito_) fluctuations. This method involves analyzing the distribution of TMRM fluorescence intensity in a model that is restricted to the mitochondrial geometry. We show that mitochondrial Ca^2+^ elevation hyperpolarizes the CM most likely caused by Ca^2+^ sensitive increase of mitochondrial tricarboxylic acid cycle (TCA) and subsequent oxidative phosphorylation (OXPHOS) activity in the cristae. Dynamic multi-parameter correlation measurements of spatial mitochondrial membrane potential gradients, ATP levels, and mitochondrial morphometrics revealed a CJ-based membrane potential overflow valve mechanism protecting the mitochondrial integrity during excessive cristae hyperpolarization.

## Introduction

The structure of mitochondria includes two membranes—the inner mitochondrial membrane (IMM) and the outer mitochondrial membrane (OMM). The IMM is divided into two compartments: (1) the crista membrane, which forms membrane folds into the mitochondrial matrix and contains complexes I, III, and IV, F_1_F_O_-ATP-synthase, and (2) the inner boundary membrane (IBM), which connects to the inner leaflet of the OMM. The crista junction (CJ) is a narrow structure that acts as a barrier for proteins and ions, separating the two compartments of the IMM^1^.

Several proteins are involved in the formation and regulation of the CJ. The mitochondrial contact site and cristae organizing system (MICOS) complex provides the scaffold and backbone of the highly curved CJ membrane shape^2^. A tight interaction between MICOS and the sorting and assembly machinery (SAM) complex creates a triangular structure that constricts the opposing sides of the IMM and the OMM^3^. While the shape of the CM is determined by the phospholipid composition and distribution of e.g. cardiolipin^3^, the local membrane potential^4^, and the F_1_F_O_-ATP-synthase dimer configuration^5^, the size of the CJ is ruled by two proteins, the mitochondrial calcium uptake 1 (MICU1)^2,4^ and optic atrophy 1 (OPA1)^4,5^. MICU1 forms oligomers that are located to the IBM and stabilize the CJ^4^. At high Ca^2+^ concentrations, the MICU1 oligomers disassemble into dimers, resulting in an open configuration that activates the opening of the CJ and allows Ca^2+^ to pass through^6^.

Recently, the regulation of the ΔΨ_mito_ was brought into context with the stability of the cristae junctions^7,8^. Stimulated emission depletion (STED) and structured illumination microscopy (SIM) was used to evaluate the question, of whether the CJ is able to separate the IMM in regard of the membrane potential^7,8^. Both studies found that the membrane potential of the IMM differs between its two compartments. The CM, harboring the proton pumps complex I, III, and IV, showed a higher (more negative) membrane potential (ΔΨ_C_) compared to the IBM (ΔΨ_IBM_). The spatial quantification of single crista depolarization indicates that the CJ seals and isolates the CM in regard of the membrane potential^7^.

In a recent study, we demonstrated that the Ca^2+^ influx pathway into the mitochondria passes the CM, which is controlled by Ca^2+^-dependent de-oligomerization of MICU1, yielding opening of the CJ^7^. Mitochondrial matrix Ca^2+^ is an important cofactor for the dehydrogenases of the of TCA-cycle, thus boosting mitochondrial ATP production^9^. In that regard, the restriction of ion diffusion through the CJ plays an important role in preventing the evasion of protons out of the cristae lumen into the intermembrane space and potentially into the cytosol^8^. Moreover, mitochondrial fission is closely associated with CJ stability and Ca^2+^ signaling. In particular, the opening of CJ, IMM constriction, and mitochondrial Ca^2+^ signals precede the fission process of mitochondria ^7,10^.

To date, super-resolution microscopy is the only possible technical solution to observe the dynamics of the mitochondrial ultrastructure in living cells^11,12^. STED and SIM microscopy were used recently to differentiate the membrane potential of mitochondrial substructures^7,8^. Nevertheless, the dynamics of the various mitochondrial membrane potential distribution between CM and IBM has not been investigated so far. For that purpose, we developed a method that builds on fluorescence intensity distribution in a model limited to mitochondrial geometry to analyze the CM/IBM membrane potential distribution in living cells. We used IMM selective and specific dyes MitoTracker™ Green FM (MTG) and tetramethylrhodamine methyl ester (TMRM) ^7,8,11,13^. Without the requirement of the transfection of genetically encoded sensors, this method allows us to track changes in membrane potential gradients over time and to correlate them with mitochondrial ATP-production and morphology. Using this method could enhance comprehension of physiological processes related to CJ, mitochondrial ATP-production, fission, apoptosis, and mitochondrial uncoupling.

## Results

### Analysis of spatial membrane potential gradients in mitochondria

We used structured illumination microscopy (SIM) to measure the spatial membrane potential gradients (SMPG) across the cross-section of mitochondria. This was done by utilizing tetramethylrhodamine methyl ester (TMRM), a dye that is sensitive to membrane potential. We utilized MitoTracker™ Green FM (MTG) as a reference. MTG accumulates in the IMM based on the potential of the mitochondrial membrane, but once it is located in the IMM, it does not respond to any changes in the membrane potential (as shown in Supplementary Fig. S1). MTG is a commonly used dye to determine mitochondrial morphology. While long term effects on cell viability were described, no short-term effects of the dye regarding cell viability ^14^ or Ca^2+^ signaling ^15^ were reported.

**Figure 1:**
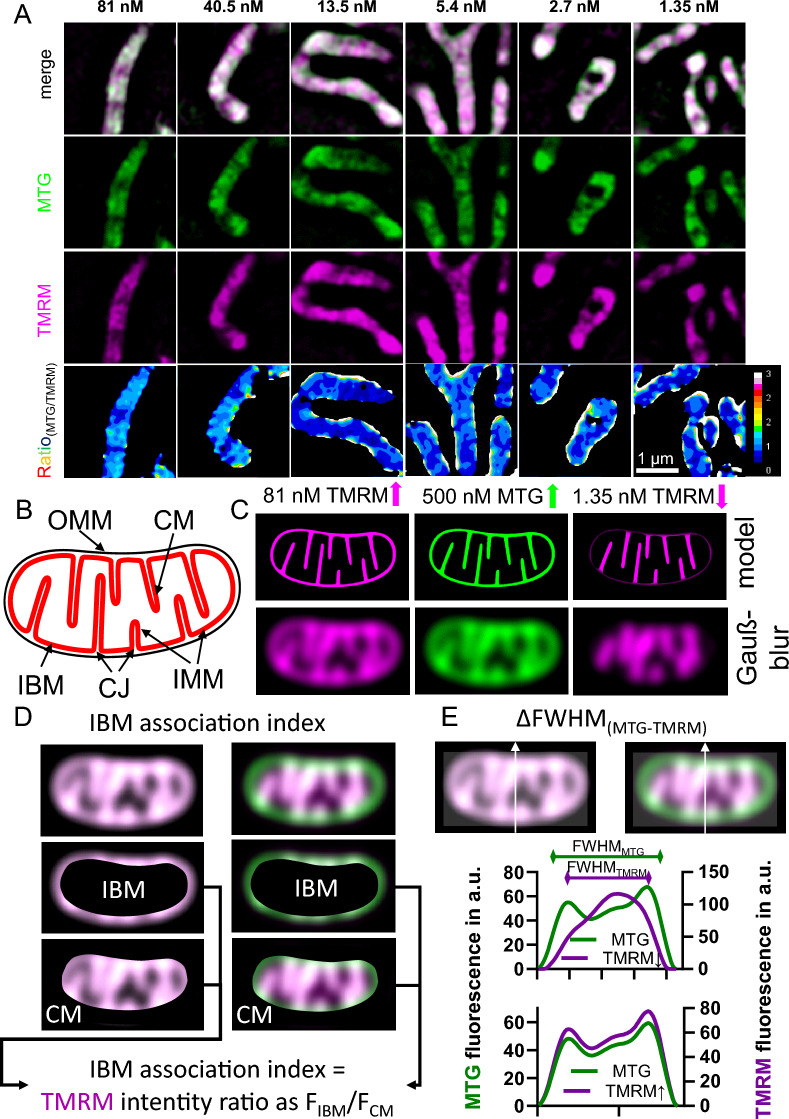
Revealing different mitochondrial membrane potentials of cristae membrane and inner boundary membrane by using two mitochondrial markers MTG and TMRM. (**A**) HeLa cells stained with 500 nM MTG and 1.35–81 nM TMRM for 1 h were imaged using dual color SIM. The ratio image (MTG/TMRM) of both channels shows increasing halo structures around mitochondria the lower the TMRM concentration. (**B**) Model of the mitochondrial morphology indicating the outer mitochondrial membrane (OMM), inner mitochondrial membrane (IMM), cristae membrane (CM), inner boundary membrane (IBM) and cristae junctions (CJ). (**C**) Model of high MTG (500 nM) and TMRM (81 nM) and low TMRM (1.35 nM) distribution throughout the IMM based on the assumption of high CM and lower IBM membrane potential and experimental data. (**D**) Schematic description of the IBM association index calculation for high (81 nM, left) and low (1.35 nM, right) TMRM concentrations. MTG serves as a stable reference for the morphological estimation of mitochondrial dimensions. (**E**) Schematic description of the ∆FWHM calculation for high (81 nM, left) and low (1.35 nM, right) TMRM concentrations. MTG serves likewise to the IBM association index calculation as a stable reference for the morphological estimation of mitochondrial dimensions.

Simultaneous dual-channel N-SIM imaging of both dyes was performed. We kept the MTG concentration constant at 500 nM while varying the TMRM concentration between 1.35 and 81 nM. Using MTG as an IMM reference marker enabled the generation of a ratio image of MTG/TMRM that illustrates the shift of TMRM fluorescence to the IBM (Fig. 1A). Notably, the distribution of TMRM depended on the TMRM affinity to the higher (more negative) membrane potential and the concentration-dependent saturation of the dye in mitochondria at high (81–40.5 nM) concentrations^7^. Low concentrations (2.7–1.35 nM) of TMRM gather in the cristae, resulting in most of the fluorescence being centered in the mitochondria's middle. However, if the concentration is high (81–40.5 nM), the cristae will become saturated, causing TMRM staining to increase relatively in the IBM (Fig. 1B, C). To distinguish the varying membrane potentials at the cristae and IBM, we analyzed the TMRM distributions based on their concentration dependence. We employed two distinct analysis methods for this purpose. (I) The IBM association index was previously used to determine the translocation of proteins between the cristae and IBM^4,7^. This index is fully automated. In short, the MTG channel is used as a spatial reference. Using an automated Otsu threshold, the boundaries of mitochondria are defined. Next, by individual shrinking and widening of boarders two regions are set (IBM and CM) in which the fluorescence intensity is measured and set into a ratio that we defined as IBM association index^4,7^. (Fig. 1D). (II) The semi-automated ∆FWHM method builds on the full width at half maximum (FWHM) of the cross-section intensity profiles of MTG and TMRM. The higher the difference/delta of the respective FWHMs of MTG and TMRM the more TMRM is accumulating in the cristae ^7^ (Fig. 1E).

Two cell lines were used in this study to verify the methodological approach of dynamic mitochondrial membrane potential distribution measurements. HeLa cell represent a strongly glycolytic cell line^16^, while EaHy-cells have a slightly stronger OXPHOS activity^17^. The similar mitochondrial morphology of both cell lines increased comparability.

**Figure 2:**
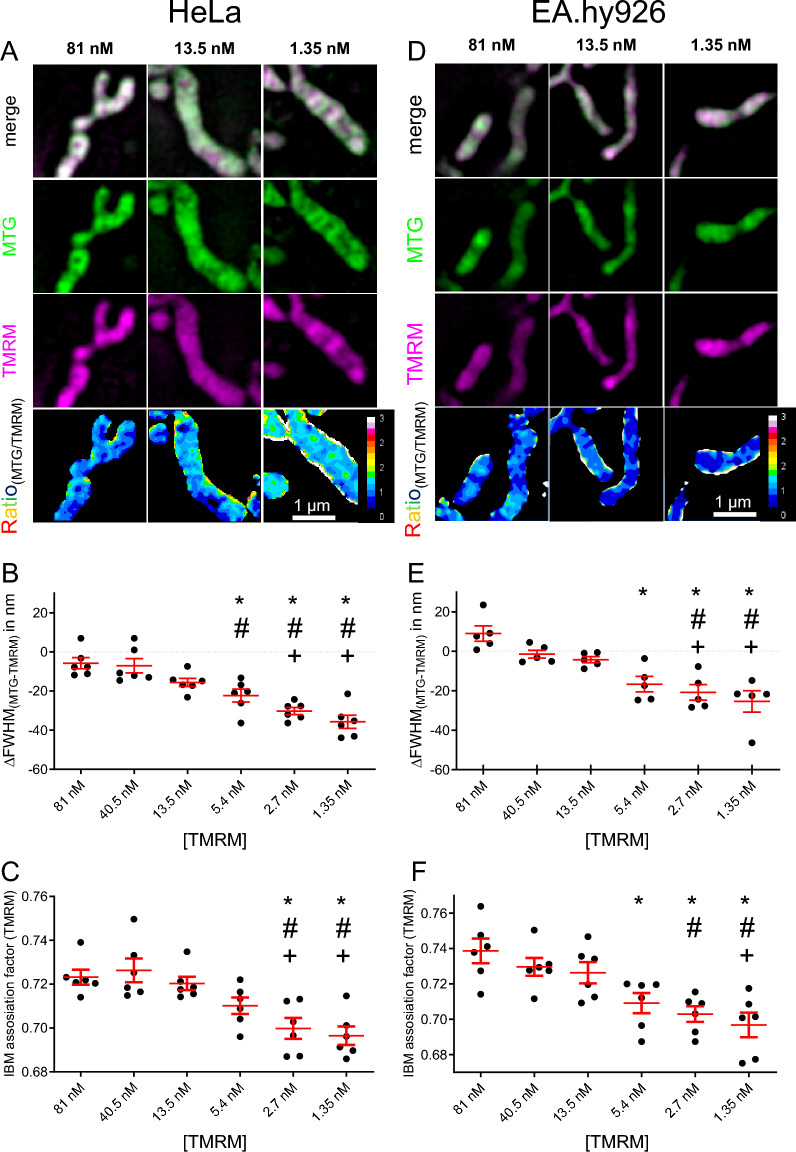
Measurement of ∆FWHM and IBM association index for HeLa and EA.hy926 cells show different membrane potential for CM and IBM. (**A**) HeLa cells stained with 500 nM MTG and 1.35, 13.5, or 81 nM TMRM for 1 h were imaged using dual color SIM. Presented are MTG (green), TMRM (magenta), merge and the ratio of MTG/TMRM. (**B**) ΔFWHM_(MTG-TMRM)_ was calculated in HeLa cells for 1.35–81 nM TMRM concentrations. (**C**) IBM association index was calculated in HeLa cells for 1.35–81 nM TMRM concentrations. (**D**) EA.hy926 cells stained with 500 nM MTG and 1.35, 13.5, or 81 nM TMRM for 1 h were imaged using dual color SIM. Presented are MTG (green), TMRM (magenta), merge and the ratio of MTG/TMRM. (**E**) ΔFWHM_(MTG-TMRM)_ was calculated in EA.hy926 cells for 1.35—81 nM TMRM concentrations. (**f**) IBM association index was calculated in EA.hy926 cells for 1.35–81 nM TMRM concentrations. Data are shown as dot plots with the mean ± SEM as red line and whiskers. Images and analyses were obtained from each 9–10 cells in 6 independent experimental days (n = 6). *P < 0.05 vs. 81 nM, ^#^P < 0.05 vs. 40.5 nM, and ^+^P < 0.05 vs. 13.5 nM TMRM conditions evaluated using one-way analysis of variance (ANOVA) with Bonferroni post hoc test.

We utilized TMRM concentrations of 1.35, 2.7, 5.4, 13.5, 40,5 or 81 nM in HeLa and EA.hy926 cells (Fig. 2A, D). We observed a distinct TMRM distribution gradient that was dependent on the concentration used. Both the ∆FWHM-based (Fig. 2B, E) and the IBM association index-based method (Fig. 2C, F) showed significant differences between high (81–13.5 nM) and low (5.4–1.35 nM) TMRM concentrations. Accordingly, we concluded that the mitochondrial IMM double staining with MTG and TMRM in combination with the IBM association index or ∆FWHM method is suitable to measure the relative difference between cristae and IBM membrane potential.

### Histamine-induced changes of membrane potential gradients

**Figure 3:**
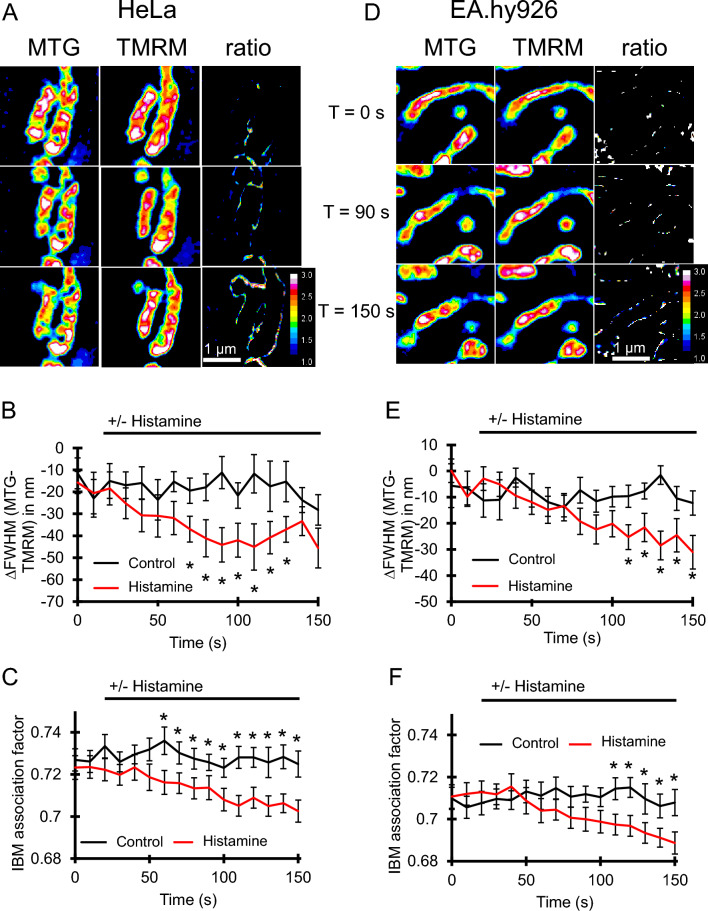
IBM association index and ∆FWHM measurements enable dynamic observation of mitochondrial membrane potential gradient during histamine stimulation. (**A**) Representative time-lapse SIM-images of HeLa mitochondria stained with MTG and TMRM. Cell were stimulated with histamine at t = 20 s. Pseudo colored MTG, TMRM and the ratio image (MTG/TMRM) are shown over time. (**B**) Quantification of ∆FWHM in HeLa cells over time with and without histamine stimulation at t = 20 s. (**C**) Quantification of the IBM association factor in HeLa cells over time with and without histamine stimulation at t = 20 s (n_control_ = 28, n_histmaine_ = 33). (**D**) Representative time-lapse SIM-images of EA.hy926 mitochondria stained with MTG and TMRM. Cell were stimulated with histamine at t = 20 s. Pseudo colored MTG, TMRM and the ratio image (MTG/TMRM) are shown over time. (**E**) Quantification of ∆FWHM in EA.hy926 cells over time with and without histamine stimulation at t = 20 s. (**F**) Quantification of the IBM association factor in EA.hy926 cells over time with and without histamine stimulation at t = 20 s. (n_control_ = 20, n_histmaine_ = 17). Data are shown as the mean ± SEM as connected line and whiskers. * P < 0.05 vs. control condition without histamine. T-test was used for evaluation of the statistical significance.

Next, we analyzed the dynamics of SMPGs after mitochondrial stimulation by elevated Ca^2+^. For that purpose, we added the IP_3_-generating agonist histamine to HeLa and EA.hy926 cells to evoke a Ca^2+^ release from the endoplasmic reticulum (ER). Both cell lines were labeled with 500 nM MTG and 13.5 nM TMRM. After histamine stimulation, a decrease in the IBM association factor and of the ∆FWHM (Supplementary Fig. S2) was measured in both cell types (HeLa cells, Fig. 3A–C; EA.hy926 cells, Fig. 3D–F), thus indicating an increase of the absolute membrane potential in the cristae relative to the IBM in response to mitochondrial Ca^2+^ uptake. We assume that the histamine-induced elevation in mitochondrial Ca^2+^ leads to an increased TCA activity^18^ that, in turn, leads to higher proton pump activity of complexes I, III, and IV found in the cristae and, thus increasing ΔΨ_C_. To test if the change of membrane potential distribution after histamine addition is caused by higher proton pump activity we used Rotenone and Antimycin A to inhibit Complex I and III, respectively. Both compounds lead to an inhibition of the histamine induced reduction of the IBM association index, indicating that the changes in mitochondrial membrane potential distribution are dependent on the proton pump activity (Supplementary Fig. S3). Interestingly, we could observe within these experiments an abrupt mitochondrial fragmentation directly after the addition of histamine, indicated by an increase in mitochondrial count and reduction of the mitochondrial aspect ratio and form factor, found in both cell types (Supplementary Fig. S4).

### ATP production is directly correlated to membrane potential gradients

**Figure 4:**
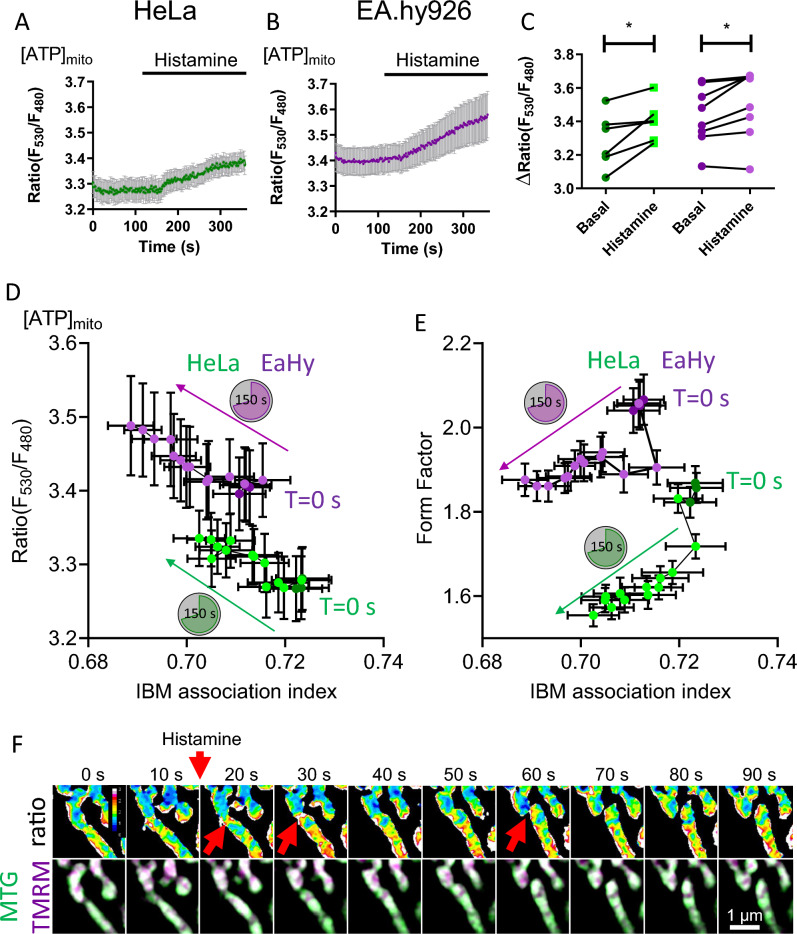
Histamine stimulation induces changes in mitochondrial membrane potential gradients followed by [ATP]_mito_ increase. (**A**) HeLa cells expressing mtAT1.03 were challenged with histamine and the response in [ATP]_mito_ was recorded. (**B**) EA.hy926 cells expressing mtAT1.03 were challenged with histamine and the response in [ATP]_mito_ was recorded. (**C**) Quantification of basal and histamine induced increase of [ATP]_mito_ in HeLa and EA.hy926 cells (n_HeLa_ = 6 , n_EA.hy926_ = 8). (**D**) The correlation of IBM association index with mitochondrial ATP is shown before and after histamine addition for HeLa (R^2^ = 0.79) and EA.hy926 (R^2^ = 0.86) cells. *T* = 0–20 s are shown as dark green (HeLa) and magenta (EA.hy926) and post treatment timepoints are shown as light green (HeLa) and light magenta (EA.hy926). (**E**) The correlation of IBM association index with mitochondrial form factor is shown before and after histamine addition for HeLa (R^2^ = 0.59) and EA.hy926 (R^2^ = 0.71) cells. *T* = 0–20 s are shown as dark green (HeLa) and magenta (EA.hy926) and post treatment timepoints are shown as light green (HeLa) and light magenta (EA.hy926). (**F**) HeLa cells labeled with TMRM and MTG were treated with histamine and imaged over time. The event shows the partial depolarization of the mitochondrion and subsequent mitochondrial fission at position. Data are shown as the mean ± SEM as connected line and whiskers. *P < 0.05 vs. control condition without histamine. Paired T-test was used for evaluation of the statistical significance.

To analyze the effects of the increase in ΔΨ_C_, we measured mitochondrial ATP production using the Förster resonance energy transfer (FRET)-based genetically encoded sensor mtAT1.03^19^. Stimulation with histamine evoked a small but significant increase in the FRET ratio signal of mtAT1.03, indicating a moderate net elevation of mitochondrial ATP concentration in both cell types as a consequence of mitochondrial Ca^2+^ uptake (Fig. 4A–C). Next, we correlated the IBM association index of the TMRM measurements with the ATP concentration after histamine addition and found a linear correlation (Fig. 4D). Based on these data, we assume that there is a direct correlation between ΔΨ_C_ with the OXPHOS-fueled ATP production. Additionally, there was a correlation between mitochondrial shape and the alteration in the distribution of membrane potential. The form factor reduced especially in the phase directly after histamine addition for both HeLa and EA.hy926 cells, indicating mitochondrial fission (Fig. 4E) (Supplementary Fig. S4). These initial fission events did not coincide with changes of TMRM distribution or ATP concentration, which occur later. This points to a series of events that happen in a chronological order. Upon ER Ca^2+^ release, mitochondrial morphology seems to be rearranged and adapted to produce more ATP through OXPHOS or protect the mitochondrial network from damage^20^. However, we have noticed instances where a local decrease or loss in membrane potential was followed by mitochondrial fission at that same location (Fig. 4F). The gradual decrease in aspect ratio and rise in the number of mitochondria over time suggest that Ca^2+^ is causing the mitochondria to swell (Supplementary Figs. S4, S5).

### Imbalance of mitochondrial membrane potential gradients by inhibition of F_1_F_O_-ATP-synthase

**Figure 5:**
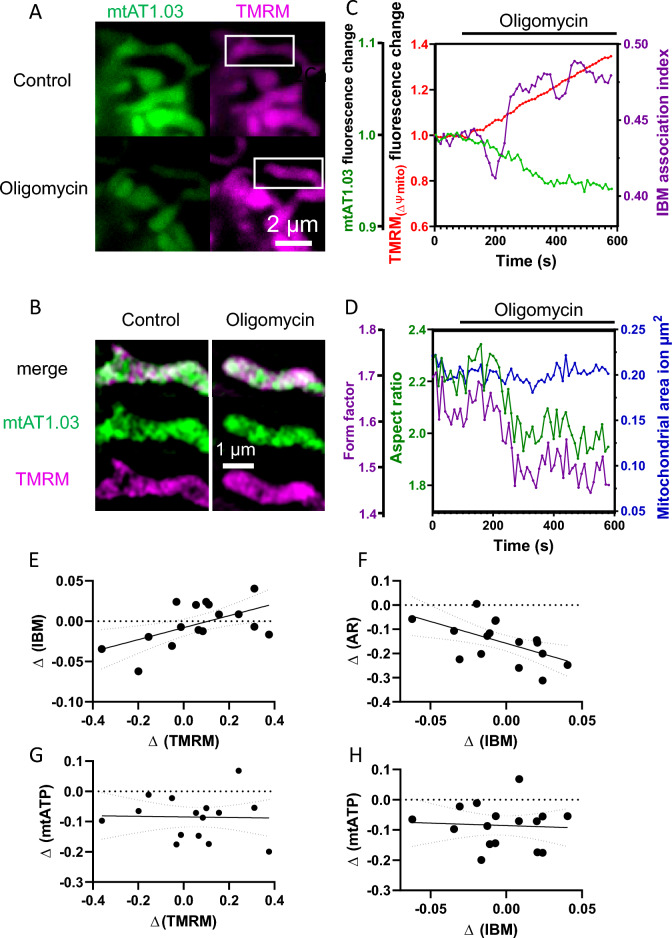
Multi variable image analysis correlates mitochondrial fission, membrane potential and membrane potential gradients. (**A**) Widefield images of mtAT1.03 transfected HeLa cell labeled with TMRM before and after addition of oligomycin. (**B**) Structured illumination microscopy image of the white rectangle in (**A**) of matrix localized mtAT1.03 and IMM localized TMRM before and after oligomycin treatment. (**C**) Analysis of the whole cell shown in (**A**) of TMRM and mtAT1.03 fluorescent intensity changes and TMRM IBM association index over time. Cell were treated with oligomycin at t = 120 s. (**D**) Analysis of the whole cell shown in a of aspect ratio, form factor and mitochondrial size over time. Cell were treated with oligomycin at t = 120 s. (**E**) Correlation of TMRM IBM association index and TMRM fluorescent intensity changes induced by oligomycin treatment (R^2^ = 0.3; p = 0.02). (**F**) Correlation of aspect ratio and IBM association index changes induced by oligomycin treatment (R^2^ = 0.31; p = 0.02). (**G**) Correlation of mtAT1.03 and TMRM fluorescent intensity changes induced by oligomycin treatment (R^2^ = 0.0007; p = 0.92). (**H**) Correlation of aspect ratio and IBM association index changes induced by oligomycin treatment (R^2^ = 0.004; p = 0.82).

To get a better understanding of the mechanisms of mitochondrial fission and CJ dynamics in regard of mitochondrial membrane potential distribution, a multi-parameter analysis of HeLa cells treated with the F_1_F_O_-ATP-synthase inhibitor oligomycin was done. Relative changes in the mitochondrial membrane potential and mitochondrial ATP levels were analyzed over time. For that purpose, the 15 recorded SIM images normally reconstructed to one super-resolved image were simply superimposed to get a widefield image of the cellular region of interest (Fig. 5A). Moreover, the super-resolution SIM images were used to determine the IBM association factor of TMRM with the matrix labeling of mtAT1.03 as a reference (Fig. 5B). After administering oligomycin, we noticed a significant increase in mitochondrial membrane potential (TMRM fluorescence intensity) and a decrease in mitochondrial ATP levels in numerous cells. Interestingly, while upon the addition of oligomycin the membrane potential and mitochondrial ATP showed a linear correlation with time, the IBM association index of TMRM showed an initial drop, followed by a rather linear rise (Fig. 5C). Simultaneous readings of the mitochondrial morphology showed decreasing form factor and aspect ratio, while the mitochondrial size was unaffected (Fig. 5D).

Interestingly, the onset of the strong increase of the IBM association index of TMRM after the initial drop was associated with the onset of changes in the morphological indicators, form factor and aspect ratio. We evaluated the correlation between changes in membrane potential and the IBM association factor, finding a weak (R^2^ = 0.3) but significant (p = 0.02) linear positive correlation (Fig. 5E). Additionally, we observed a slight (R^2^ = 0.31) but significant (p = 0.02) negative correlation between changes in aspect ratio and the IBM association factor (Fig. 5F). These findings suggest that changes in mitochondrial morphology are influenced by membrane potential and its distribution on the IMM. However, we did not find any correlation between mitochondrial ΔATP levels and membrane potential changes or membrane potential gradients changes (Fig. 5G, H).

## Discussion

Processes like mitochondrial Ca^2+^ uptake or protein import show distinct locations of action within the IMM^7^. The inhomogeneous localization and organization of complexes I, II, III and IV and the F_1_F_O_-ATP-synthase inside the cristae^21^ indicate a similar inhomogeneous distributed membrane potential. In the present study, we adapted a previously used method^4,7^ to correlate the dynamics of mitochondrial membrane potential gradients between the CM and IBM during physiological stimuli with the status of mitochondrial bioenergetics. Using increasing concentrations of the membrane potential-dependent IMM marker TMRM and the membrane potential-independent dye MTG, we could discriminate two different mitochondrial membrane potentials, belonging to the CM (ΔΨ_C_) and IBM (ΔΨ_IBM_). Both analysis techniques used to identify membrane potential gradients in mitochondria, the IBM association index and the ΔFWHM, rely on the assumption of a barrel-shaped mitochondrion with clear geometrical borders.

It was shown that TMRM, TMRE and MTG are binding specific to the IMM^4,8,12^. More precise, TMRE binds predominantly to the inner leaflet of the IMM^13^, which could be directly applied to TMRM as well, due to the high structural similarity. The phospholipid composition of CM and IBM might have an influence on TMRM and MTG distribution. Further studies should be conducted to analyze this potential pitfall, which is important for all membrane potential related measurements, especially in regard of microdomains within mitochondria.

The mitochondrial diameter varies between 200 and 300 nm. Thus, conventional confocal microscopy might not be able to resolve in all cases the mitochondrial cross-section intensity distribution without being limited by the resolution capabilities, thus the use of super-resolution microscopy appears a prerequisite. However, even due to the resolution limits of super-resolution techniques, like SIM, a clear discrimination between single crista is not possible. Because several crista might superimpose within the resolution limit of SIM of app. 100–140 nm, rendering a Nernst-equation-based analysis of TMRM fluorescence intensity unreliable. Therefore, we focused on the average distribution between CM and IBM, which is achievable with our dual N-SIM, or comparable microscopy techniques with similar properties concerning image resolution, like ZEISS Airyscan^8,13^, Yokogawa SoRa^22^, or CrestOptics DeepSIM^23^. Accordingly, the analysis methods presented herein are applicable to these microscopy techniques without major adaptations.

In the present study, we measured the effects of histamine stimulation on the dynamics of spatial mitochondrial membrane potential distribution. Hence, we correlate these findings with mitochondrial morphology readouts, and the measurement of mitochondrial ATP concentrations. Our results indicate that mitochondria undergo rapid morphological adaptations after exposure to high Ca^2+^ approaching from the ER, which precedes the Ca^2+^-mediated metabolic boost on the TCA cycle and ATP production. During ER-Ca^2+^ release, the cristae junctions open in a Ca^2+^-dependent manner, in a process mediated by MICU1. This results in a short-term increase in Ca^2+^ influx in regions of the mitochondrial-associated ER membranes (MAMs)^7^. It is known that Ca^2+^ signaling leads to mitochondrial fragmentation^20^ to protect mitochondria from Ca^2+^ overload. Our observation of local mitochondrial membrane depolarization ahead of the initiation of fission events at the same localization, emphasizes a causality between Ca^2+^ mediated CJ opening^7^, cristae depolarization, and subsequent mitochondrial fission in a predefined location^10^. A similar mechanism might be involved in the mitochondrial fission-driven separation of damaged mitochondria with disturbed F_1_F_O_-ATP-synthase or OXPHOS metabolism^24^, resulting in an opening of the CJ and subsequent fission and provision of mitochondria to mitophagy. After such Ca^2+^-induced fission, the membrane potential clusters in the cristae of the remaining mitochondrion may recover because of a closed CJ configuration and increased TCA and OXPHOS activity. Accordingly, CJ plays an important role in mitochondrial organization because it isolates the CM from the IBM, keeping the mitochondrial membrane potential confined for the use of ATP production^7,8^.

The closure or opening of the junction between mitochondria and the ER (known as the MAMs facing the CJ) depends on how long and how much Ca^2+^ is elevated. When there is a moderate amount of Ca^2+^, the junction closes quickly, maintaining ΔΨ_C_, and the mitochondrial processes of OXPHOS and ATP production^7^. However, when there is too much Ca^2+^, the junction stays open, leading to fission which helps to protect the mitochondria network from damage.

Using simultaneous measurements of membrane potential, membrane potential distribution, mitochondrial ATP levels, and the morphological status of mitochondria, a holistic impression of the mitochondria's metabolic status can be achieved. Inhibition of the F_1_F_O_-ATP-synthase in HeLa cells by oligomycin shows their highly heterogeneous metabolic state^16^. A stronger membrane potential and a decrease in mitochondrial ATP concentrations indicate the forward mode of the F_1_F_O_-ATP-synthase, while a drop of membrane potential points to a reverse mode F_1_F_O_-ATP-synthase, assisting the OXPHOS to maintain the mitochondrial membrane potential^16,25^. Oligomycin leads initially to an increase of ΔΨ_C_, followed by a strong increase of ΔΨ_IBM_, which is associated with mitochondrial swelling and fragmentation. We hypothesize that the block of F_1_F_O_-ATPsynthase results in an accumulation of protons in the cristae which exceed the proton-buffering capacity of cardiolipin or other phospholipids in the IMM^26^. This may lead, by a so far unknown process, to the opening of the CJ resulting in the harmonization of ΔΨ_C_ and ΔΨ_IBM_. A potential interaction partner of cardiolipin stabilizing the CJ is MICU1^4,27^. Our observations might also shed light on reports of increased ROS production in cells with knockdown of MIC10 or MIC60^28,29^. Both proteins are components of the MICOS complex. Knockout of MIC10 or MIC60 leads to onion-like cristae, lacking CJ almost entirely^30^. Accordingly, CM hyperpolarization leading to ROS could be inhibited by an overflow valve mechanism through the CJ representing a preventive uncoupling mechanism to avoid mitochondrial damage through excessive ROS production. The opening of the CJ also leads to unrestricted access of ions like Ca^2+^ into the cristae lumen, subsequently leading to mitochondrial swelling and fragmentation^31^.

In the present work, we describe a technique that allows the correlation of the distribution of membrane potential within the IMM compartments (CM, IBM) with mitochondrial morphometrics and bioenergetics. The use of membrane permeable dyes makes transfection unnecessary which may be a benefit in fresh isolated cells and any cell type that cannot be transfected. Our work shows for the first time dynamic measurements of spatial membrane potential distribution in intact cells and not snapshot analyses like in the previous or others’ work ^7,8,13^. The described technic of IBM association index and ΔFWHM measurements are adaptable and applicable to a wide variety of super- and high-resolution technics. We discovered first signs of a protective mechanism by the opening of cristae junctions against enhanced ROS production due to excessive cristae hyperpolarization.

The correlation of membrane potential distribution with mitochondrial morphometrics and bioenergetics depends on the duration and/or amplitude of the Ca^2+^ elevation within the MAMs facing the CJ. At moderate Ca^2+^ challenge, the CJ rapidly closes, thus preserving ΔΨ_C_, OXPHOS, and ATP production. Under conditions of excessive Ca^2+^ stress, the CJ stays open and, thus, initiates fission, which protects the mitochondrial continuum for damage.

## Methods

### Structured illumination microscopy (SIM)

Single and dual camera SIM imaging. The SIM-setup used is composed of a 405 nm, 488 nm, 515 nm, 532 nm and a 561 nm excitation laser introduced at the back focal plane inside the SIM-box with a multimodal optical fiber. For super-resolution, a CFI SR Apochromat TIRF 100x-oil (NA 1.49) objective was mounted on a Nikon-Structured Illumination Microscopy (N-SIM^®^, Nikon, Austria) System with standard wide field and SIM filter sets and equipped with two Andor iXon3^®^ EMCCD cameras mounted to a Two Camera Imaging Adapter (Nikon Austria, Vienna, Austria). At the bottom port a third CCD-camera (CoolSNAP HQ2, Photometrics, Tucson, USA) is mounted for wide-field imaging. For calibration and reconstruction of SIM images the Nikon software (NIS-Elements AR 4.51.00 64-bit, Nikon, Austria) was used. Reconstruction was permanently performed with the same robust setting to avoid artefact generation and ensures reproducibility with a small loss of resolution of 10% compared to most sensitive and rigorous reconstruction settings. Microscopy setup adjustments were done as described elsewhere^4^.

### Cell culture

HeLa (ATCC, CCL-2.2™) and EA.hy926 (ATCC, CRL-2922™) cells were seeded on 1.5H high precision glass cover slips (Marienfeld-Superior, Lauda-Königshofen, Germany) and cultured in DMEM (D5523, Sigma-Aldrich, Darmstadt, Germany) containing 10% FCS, penicillin (100 U/ml), streptomycin (100 µg/ml) and amphotericin B (1.25 µg/ml) (Gibco™, Thermo Fisher Scientific, Vienna, Austria) in a humidified incubator (37 °C, 5% CO_2_/95% air). Origin of cells was confirmed via STR-profiling by the cell culture facility of the Center of Medical Research (ZMF, Graz, Austria).

### Labeling with MitoTracker™ Green FM and tetramethylrhodamine methyl ester (TMRM)

Cells were washed once with loading-buffer containing in mM: 2 CaCl_2_, 135 NaCl, 5 KCl, 1 MgCl_2_, 1 HEPES, 2.6 NaHCO_3_, 0.44 KH_2_PO_4_, 0.34 Na_2_HPO_4_, 10 D-glucose (Carl Roth, Karlsruhe, Germany), 0.1% vitamins, 0.2% essential amino acids and 1% penicillin/streptomycin at pH 7.4. Cells were incubated in loading-buffer containing 81, 40.5, 13.5, 5.4, 2.7 or 1.35 nM TMRM (tetramethylrhodamine methyl ester, Invitrogen™) and 500 nM MitoTracker™ Green FM for 60 min. As TMRM might degrade over time in storage, TMRM concentrations were measured regularly. Therefore, after TMRM was dissolved in methanol the absorption at 550 nm was measured and the concentration calculated using Eq. 1.

1 $$c=A/\left(\varepsilon \cdot l\right)$$

(*c*) is the molar concentration of TMRM, (*A*) the absorption of TMRM at 550 nm, (*ε*) is the specific extinction coefficient of TMRM at 550 nm in methanol and (*l*) is the pathlength the light has to path through the TMRM solution.

### Cristae membrane and inner boundary membrane potential separation by FWHM

Hela cells were stained with different concentration of TMRM ranging from 1.35 to 81 nM and 500 nM Mitotracker Green FM (MTG). After the staining procedure the cells were kept in loading-buffer containing the respective concentrations of TMRM and imaged with dual color 3D-SIM. To compensate for intensity differences for TMRM acquisition the laser power was adjusted to match image intensity histograms of different TMRM concentrations. Post imaging, the recorded data were background corrected using an ImageJ^32^ Plugin (Mosaic Suite, background subtractor, NIH) with a sliding rectangle diameter of 50 pixel. Intensity line plot of mitochondrial MTG and TMRM fluorescence were manually measured with a width of 50 pixel (1.6 µm). The FWHM (full width at half maximum) of MTG and TMRM fluorescence distributions was measured using linear interpolation to gain subpixel information. Subtraction of FWHM_MTG_ form FWHM_TMRM_ results in ∆FWHM.

### Cristae membrane and inner boundary membrane potential separation by IBM association index

The IBM association factor of TMRM was calculated as described elsewhere^4^. In short, images were subjected to background subtraction (Mosaic Suite, background substractor, NIH) with a sliding rectangle diameter of 50 pixel. The reference channel (mtAT1.03 or MTG) was Otsu^33^ auto thresholded and further dilated and eroded in two independent subsets. 1 erosion and 2 dilation iterations were used. Pixel-wise subtraction of the erosion reference of the dilated reference image yields in a hollow structure, used as a mask to measure the mean intensity in the mitochondrial periphery or IBM related area in the object channel. The erosion reference served as a mask to measure the bulk or cristae mean fluorescence intensity. The ratio of IBM/CM mean intensity is a value to estimate changes of the object label distribution inside a mitochondrion, which is referred to as the IBM association index. The higher the ratio value the higher the distribution of protein label in the IBM. For image analysis, the freeware program ImageJ was used^32^.

### Conversion of N-SIM into widefield images

The imaging process of 3D structured illumination microscopy using a N-SIM (Nikon, Austria) involves a sequence of 15 images with different illumination patterns of the sample. The overlay of these 15 images gains a widefield image, which can be used for conventional intensiometric measurements, like TMRM or mtAT1.03 fluorescence. Thus, we gain information about global mitochondrial membrane potential or ATP production, respectively. The conversion was performed using a costume ImageJ macro^32^.

### Morphological analysis of mitochondria in 2D-SIM images

3D-SIM and time-lapsed images of, TMRM, MTG, or mtAT1.03 were used for morphological analysis. Images were background corrected with an ImageJ Plugin (Mosaic Suite, background subtractor, NIH) and the subsequent binarization was done using an Otsu^33^ auto threshold. The ImageJ particle analyzer^32^ was used to extract the mitochondrial count (*c*), area (*a*), perimeter (*p*), minor (*x*) and major (*y*) axes of the mitochondria. Aspect ratio (*AR*) was determined by Eq. (2):

2 $$AR=\frac{y}{x}$$

The form factor (*FF*) was determined by Eq. (3):

3 $$FF=\frac{{p}^{2}}{4\pi \cdot a}$$

### Mitochondrial ATP production

Mitochondrial ATP in EA.hy926 and HeLa cells was measured using a FRET-based genetically encoded mitochondrial matrix targeted AT1.03 biosensor^19^ (gift from Hiromi Imamura, Kyoto University, Japan). Mitochondrial ATP production upon stimulation with histamine (100 µM) was performed on an Olympus IX73 inverted microscope. The microscope is equipped with an UApoN340 40× oil immersion objective (Olympus, Japan) and a CCD Retiga R1 camera (Q-imaging, Canada). The ATP sensor was excited with 455 nm LED (LedHUB^®^, Omnicron, Germany) and emission was collected at 480 nm and 530 nm using a CFP/YFP/RFP filter set and 505dcxr beam-splitter (Semrock, USA). During the measurements, cells were continuously perfused with a physiological buffer (2 mM Ca2 + , 135 mM NaCl, 1 mM MgCl2, 5 mM KCl, 10 mM HEPES, 10 mM glucose, pH adjusted to 7.4) using a gravity-based perfusion system (NGFI, Graz, Austria). Data acquisition and control of the microscope were done using Visiview 4.2.01 (Visitron, Germany).

### Statistical analysis and reproducibility

Each exact n value and the number of independent experiments is indicated in each figure legend. Statistical analysis was performed using the GraphPad Prism software version 5.04 (GraphPad Software, San Diego, CA, USA) or Microsoft Excel (Microsoft Office 2013). Analysis of variance (ANOVA) with Bonferroni post hoc test and t-test were used for evaluation of the statistical significance. P < 0.05 was defined to be significant. At least three experiments on three different days were performed for each experimental setup.

### Supplementary Information

Supplementary Figures.

## Data Availability

The datasets generated during and/or analysed during the current study are available from the corresponding author on reasonable request.
