# Supplementary material for: Implications of mitochondrial membrane potential gradients on signaling and ATP production analyzed by correlative multi-parameter microscopy
Source: Sci Rep. 2024 Jun 26;14:14784. doi: 10.1038/s41598-024-65595-z (PMC11208492; doi:10.1038/s41598-024-65595-z)
Supplement: Supplementary file 1 — Supplementary Figures. [file 41598_2024_65595_MOESM1_ESM.pdf]

Supplementary figure 1

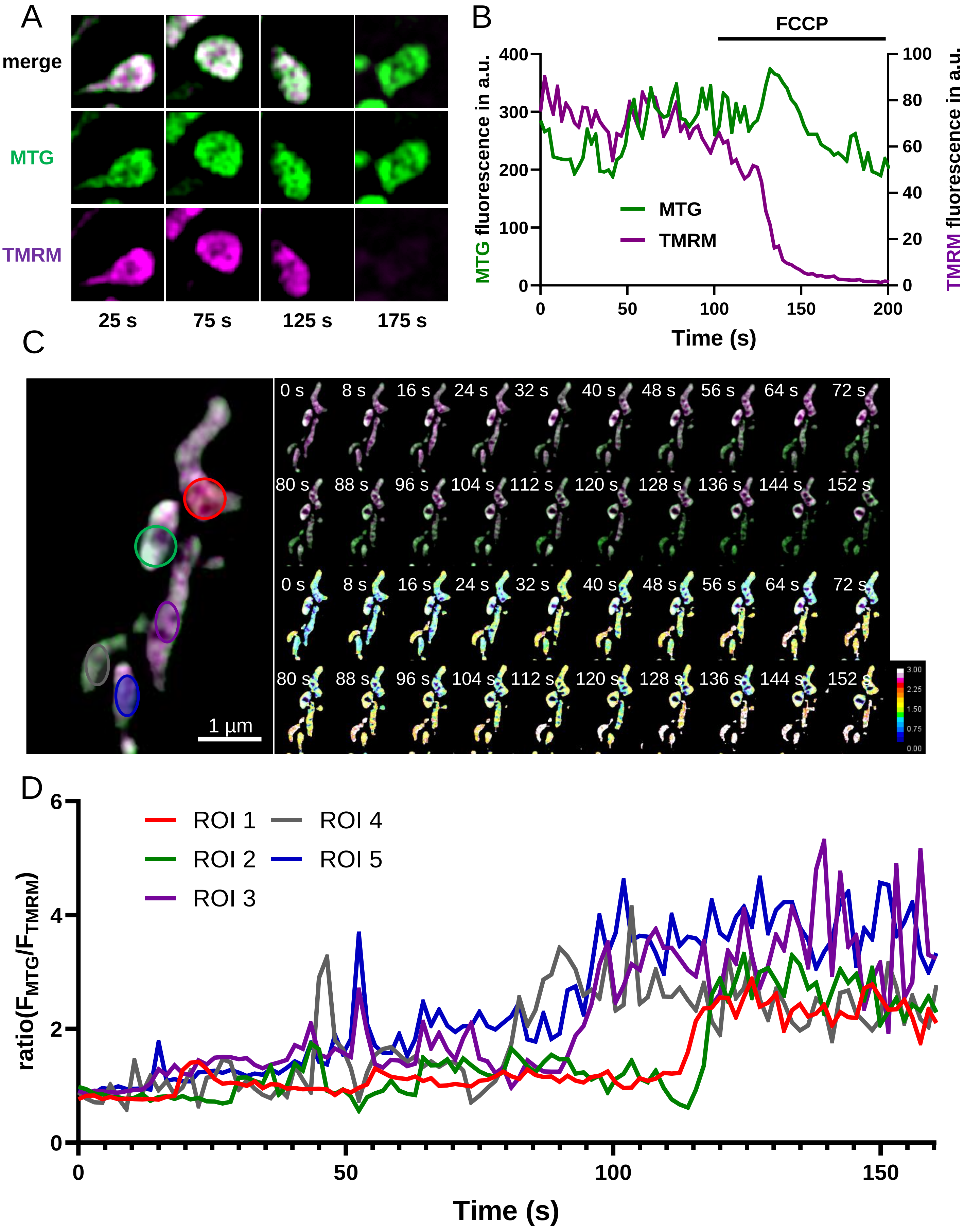

Supplementary figure 1: Membrane potential independent binding of Mitotracker green FM **to the inner mitochondrial membrane.** Mitotracker Green FM (MTG) is membrane potential independent within the mitochondria. a) Shown is a single mitochondrion over time of HeLa cells stained with MTG and TMRM and treated with FCCP at t = 100 s. b) fluorescence intensity plot of MTG and TMRM of the mitochondrion shown in a) over time. c) HeLa cell labeled with MTG and TMRM were subjected to high intensity laser illumination to induce flickering. Over time, TMRM fluorescence starts to flicker indicating temporal membrane potential loss while MTG does not show flickering. d) Ratio of MTG/TMRM fluorescence over time of the color-coded regions of interest (ROI) in c). Membrane potential loss and recovery can be monitored using the ratio.

Supplementary figure 2

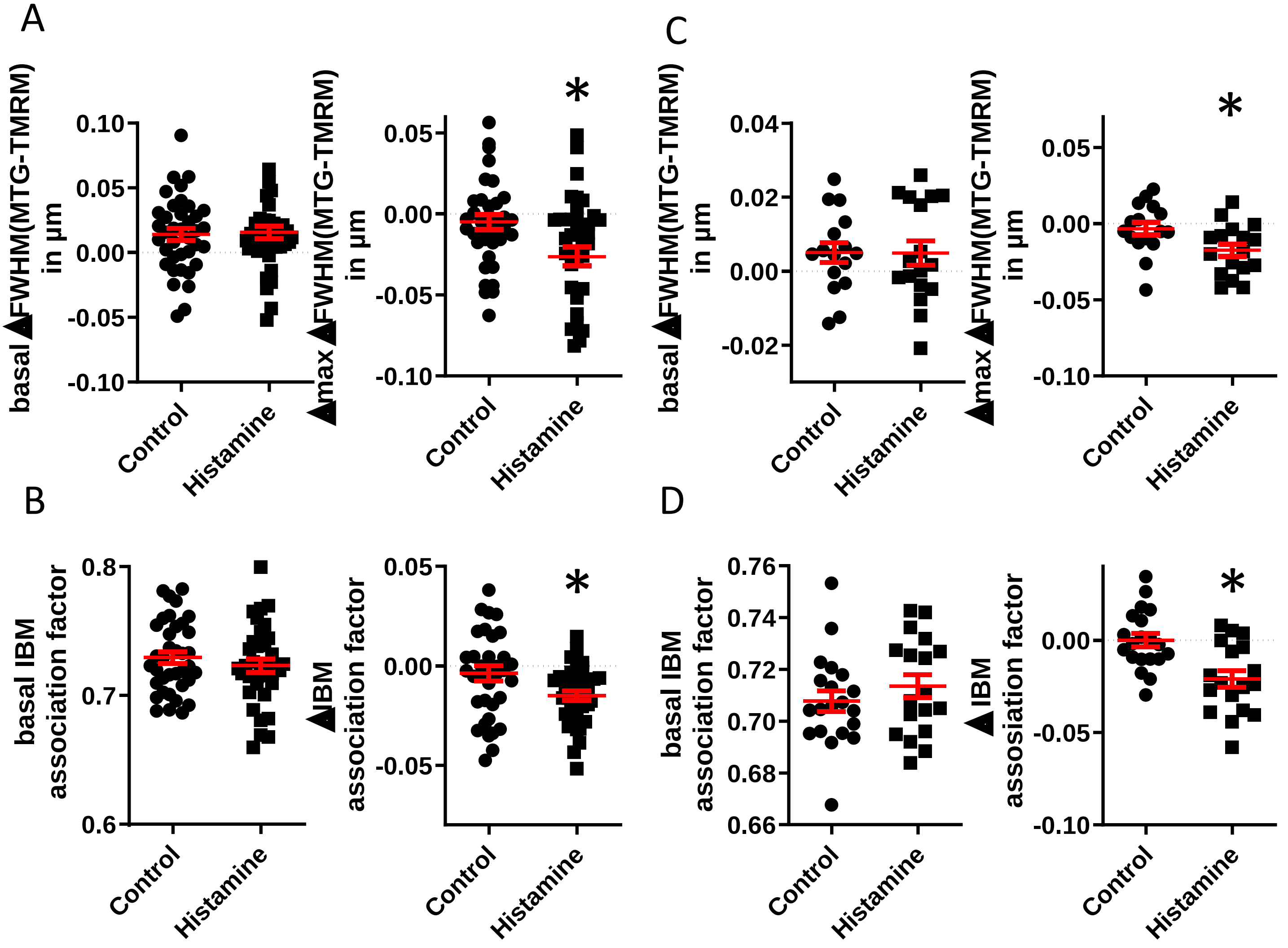

Supplementary figure 2: Statistical evaluation of basal ΔFWHM and IBM association factor and the respective dynamic changes after addition of histamine. a) Quantification of the basal and ΔFWHM in HeLa cells over time with and without histamine stimulation at t = 20 s. b) Quantification of the basal and ΔIBM association factor in HeLa cells over time with and without histamine stimulation at t = 20 s. c) Quantification of the basal and ΔFWHM in EA.hy926 cells over time with and without histamine stimulation at t = 20 s. d) Quantification of the basal and ΔIBM association factor in EA.hy926 cells over time with and without histamine stimulation at t = 20 s.

Supplementary figure 3

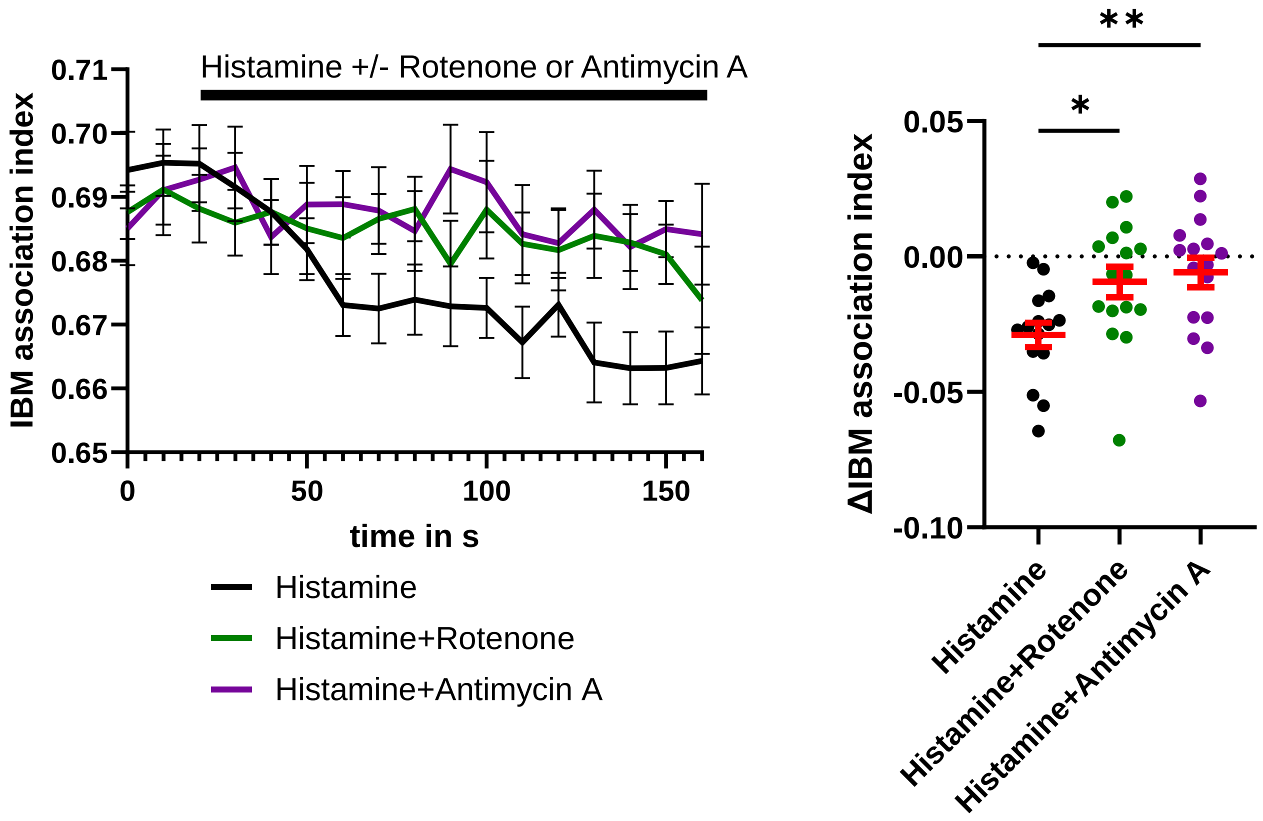

Supplementary figure 3: Rotenone and Antimycin A inhibit mitochondrial Ca^{2+} induced changes of membrane potential gradients. HeLa cells were stained with 500 nM Mitotracker Green TM and 13.5 nM TMRM. IBM association factor was quantified over time with stimulation at t = 20 s with 100 µM histamine with or without 2 µM Rotenone or 2 µM Antimycin A ($n_{Histamine}$ = 15, $n_{Histmaine+Rotenone}$ = 16, $n_{Histmaine+Antimycin\ A}$ = 16). The ΔIBM association index was calculated as the difference between basal IBM association index and the average IBM association index 110 – 130s after stimulation. * = p<0.05; ** = p<0.01 tested with ANOVA and Tukey´s multiple comparison test

Supplementary figure 4

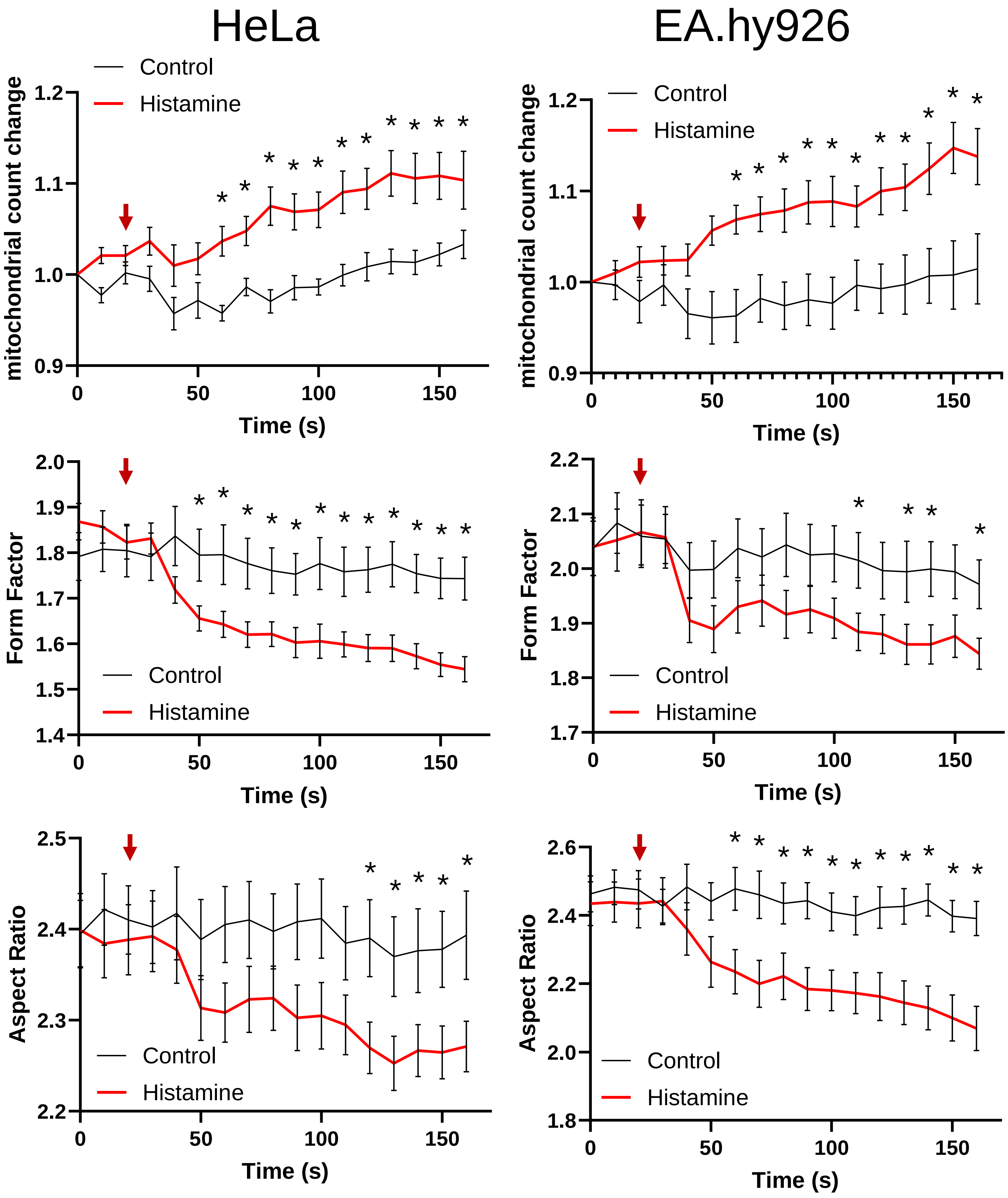

Supplementary figure 4: Dynamic mitochondrial morphological changes with and without addition of histamine. HeLa and EA.hy926 cell were labeled with MTG and TMRM. Using the MTG channel, mitochondrial form factor, aspect ratio and the change of mitochondrial count over time were measured with and without the addition of histamine at t = 20 s, indicated with a red arrow.

Supplementary figure 5

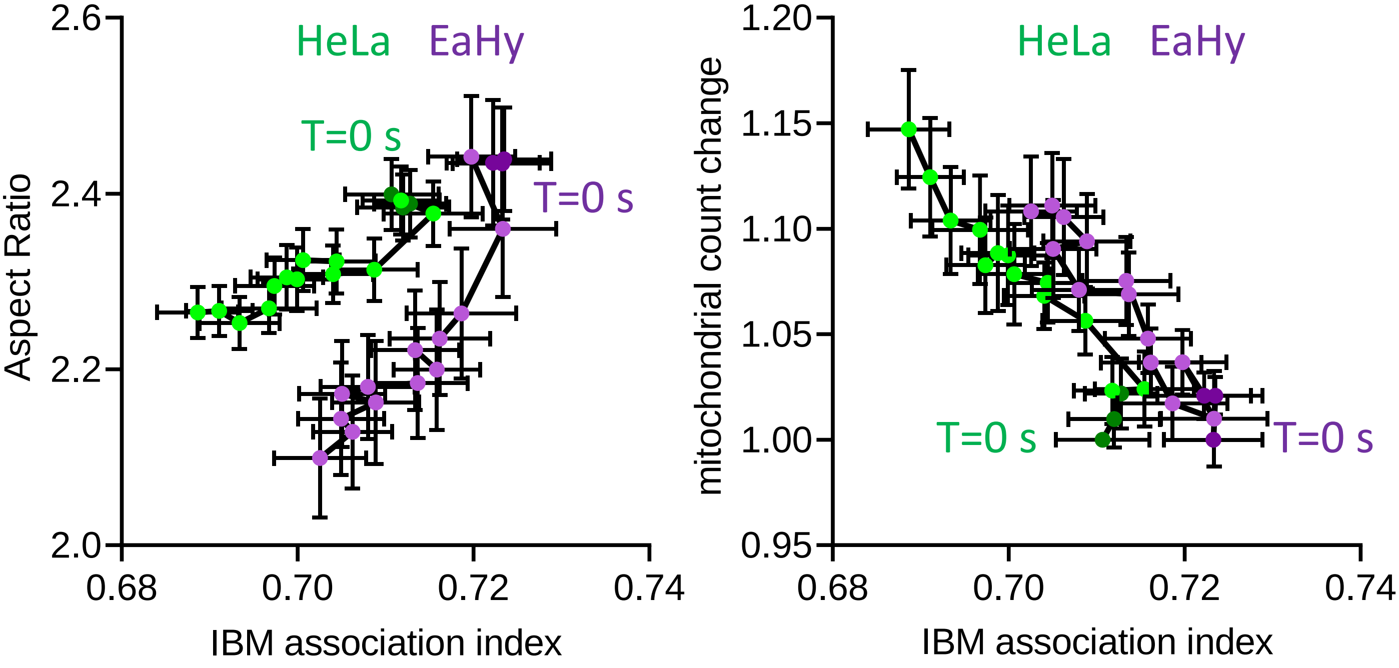

Supplementary figure 5: Correlation of IBM association factor of TMRM with mitochondrial aspect ratio and mitochondrial count. The correlation of IBM association index with mitochondrial aspect ratio is shown before and after histamine addition for HeLa and EA.hy926 cells. *T* = 0-20 s are shown as dark green (HeLa) and magenta (EA.hy926) and post treatment timepoints are shown as light green (HeLa) and light magenta (EA.hy926). The correlation of IBM association index with the relative change of mitochondrial count is shown before and after histamine addition for HeLa and EA.hy926 cells. *T* = 0-20 s are shown as dark green (HeLa) and magenta (EA.hy926) and post treatment timepoints are shown as light green (HeLa) and light magenta (EA.hy926).
